# Correction: Recent advances in surface plasmon-driven catalytic reactions

**DOI:** 10.1039/c8ra90070a

**Published:** 2018-08-24

**Authors:** Xin Ren, En Cao, Weihua Lin, Yuzhi Song, Wenjie Liang, Jingang Wang

**Affiliations:** School of Physics and Electronics, Shandong Normal University Jinan 250014 China yzsong@sdnu.edu.cn; Beijing Key Laboratory for Magneto-Photoelectrical Composite and Interface Science, School of Mathematics and Physics, University of Science and Technology Beijing Beijing 100083 P. R. China; Beijing National Laboratory for Condensed Matter Physics, Institute of Physics, Chinese Academy of Sciences Beijing 100190 P. R. China; Department of Physics, Liaoning University Shenyang 110036 P. R. China jingang_wang@sau.edu.cn

## Abstract

Correction for ‘Recent advances in surface plasmon-driven catalytic reactions’ by Xin Ren *et al.*, *RSC Adv.*, 2017, **7**, 31189–31203.

The authors regret that there was an error in one of the acknowledgements in the original article. The correct funding information for the National Basic Research Program of China is grant number 2016YFA0200800. Wenjie Liang’s name was also spelled incorrectly in the original article. The correct author names are as presented above.

The Royal Society of Chemistry apologises for these errors and any consequent inconvenience to authors and readers.

## Supplementary Material

